# Carcinoma of unknown primary (CUP): an update for histopathologists

**DOI:** 10.1007/s10555-023-10101-6

**Published:** 2023-07-03

**Authors:** Katie Beauchamp, Bruce Moran, Timothy O’Brien, Donal Brennan, John Crown, Kieran Sheahan, Maura Bríd Cotter

**Affiliations:** 1 Department of Histopathology, St Vincent’s University Hospital, Elm Park, Dublin 4, Ireland; 2 Department of Medical Oncology, Mater Misericordiae University Hospital, Dublin 7, Ireland; 3 Systems Biology Ireland, UCD School of Medicine, Belfield, Dublin4, Ireland; 4 UCD Gynaecological Oncology Group, St Vincent’s University Hospital, Elm Park, Dublin 4, Ireland; 5 Department of Medical Oncology, St Vincent’s University Hospital, Elm Park, Dublin 4, Ireland

**Keywords:** Carcinoma of unknown primary, Metastatic, Morphology, Pitfalls, Immunohistochemistry, Molecular pathology

## Abstract

Carcinoma of unknown primary (CUP) is a heterogeneous group of metastatic cancers in which the site of origin is not identifiable. These carcinomas have a poor outcome due to their late presentation with metastatic disease, difficulty in identifying the origin and delay in treatment. The aim of the pathologist is to broadly classify and subtype the cancer and, where possible, to confirm the likely primary site as this information best predicts patient outcome and guides treatment. In this review, we provide histopathologists with diagnostic practice points which contribute to identifying the primary origin in such cases. We present the current clinical evaluation and management from the point of view of the oncologist. We discuss the role of the pathologist in the diagnostic pathway including the control of pre-analytical conditions, assessment of sample adequacy, diagnosis of cancer including diagnostic pitfalls, and evaluation of prognostic and predictive markers. An integrated diagnostic report is ideal in cases of CUP, with results discussed at a forum such as a molecular tumour board and matched with targeted treatment. This highly specialized evolving area ultimately leads to personalized oncology and potentially improved outcomes for patients.

## Introduction

The majority of patients with cancer present with a clearly defined primary malignancy that manifests with typical local or metastatic symptoms. However, 10–15% present with metastatic disease from the outset rather than with a primary tumour. In about two-thirds of these, the primary site is identified during clinical investigation or over time; however, in the remaining third, the primary site is never found, termed carcinoma of unknown primary origin (CUP) [1, 2]. CUP represents a morphological, immunohistochemical and molecular diagnostic challenge in surgical pathology. In this review targeted at histopathologists, we provide practical diagnostic practice points. We also recognize that the current oncological paradigm is focussed on the therapeutic options available to CUP patients. Increasingly, this involves the use of predictive biomarkers using additional immunohistochemistry and molecular assays beyond those traditionally used to detect the primary site.

## Tissue processing and histopathology review

**Fig. 1:**
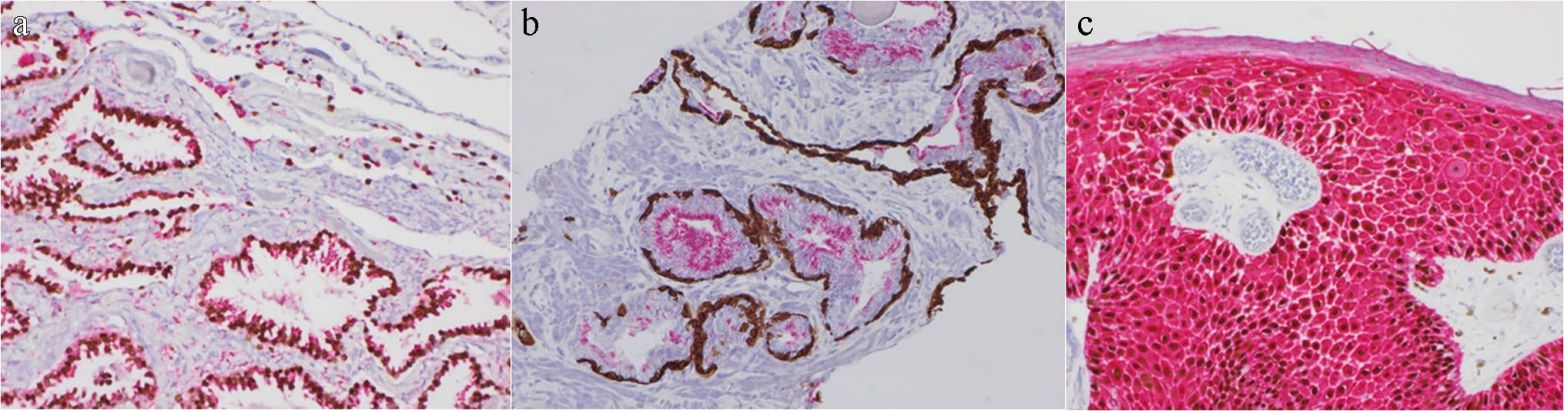
Examples of antibody cocktails **a** TTF-1/Napsin A in the lung tumour, **b** P63/34BE12 and AMACR in the prostate, and **c** P63 and CK5/6 in the skin

Initial clinical presentation varies depending on the organs involved by metastatic disease, and therefore tissue samples may be submitted from any anatomic site in a case of suspected CUP, including endoscopic biopsies, small CT/ultrasound-guided biopsies, and even smaller cytology samples. One of the main challenges pathologists face today is that samples are becoming less and less invasive resulting in smaller specimens. At the same time, advances in therapeutics have led to increased numbers and complexity of tests required from the same piece of tissue including biomarkers for risk, prognosis, and response. Therefore, the likelihood of CUP should be communicated as soon as possible so appropriate measures can be taken prior to tissue processing. In a suspected case, the optimal approach is to embed the entire biopsy, and if there are multiple fragments, they should be embedded in separate cassettes to ensure maximum diagnostic yield and to avoid the need for the patient to undergo a second biopsy. Upfront serial spare sections should be retained for use of a select immunopanel while conserving the remainder for molecular studies [3]. Antibody cocktails should also be used where possible, where two/more antibodies are placed on the same slide rather than one to conserve tissue (Fig. 1).

**Fig. 2:**
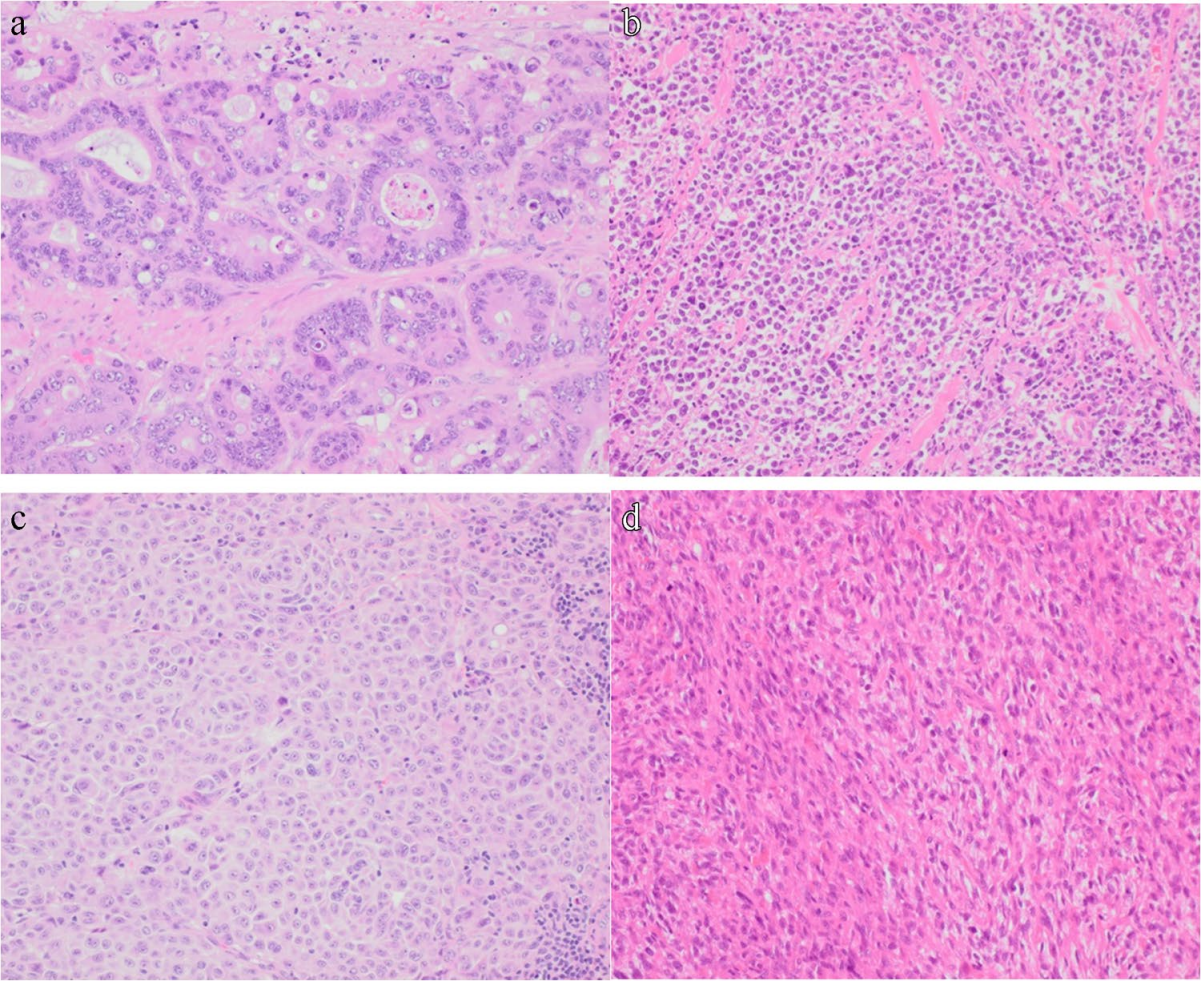
Careful morphological analysis performed on H&E can differentiate an **a** adenocarcinoma with a glandular growth pattern from non-carcinoma tumour subtypes like **b** a lymphoma composed of dyscohesive sheets of neoplastic cells within minimal cytoplasm, **c** a melanoma comprising sheets of epithelioid cells with abundant cytoplasm and prominent nucleoli, and **d** a sarcoma with spindled morphology

If malignancy is identified on biopsy, the amount of tumour tissue and amount of necrosis must be confirmed to conclude adequacy for further studies. Percentage tumour within the sample is particularly relevant if molecular studies are to be carried out, as if too small, a somatic mutation will be difficult to find as it will be overwhelmed by the DNA of background non-neoplastic cells. Next, careful morphological analysis is performed which can rule out non-carcinoma subtypes like melanoma, lymphoma, and sarcoma (Fig. 2) with or without the aid of limited immunohistochemistry such as S100, CD45, and a negative AE1/AE3 respectively while preserving tissue for further workup [3]. The majority of these tumours are broadly subtyped as metastatic adenocarcinoma (60%), squamous cell (5%), and neuroendocrine carcinoma (2%) or are poorly or undifferentiated (30%) [3]. Clearly, oncological management will be influenced by the lineage of differentiation, and Table 1 outlines the biomarkers used in some of these distinctions. The high grade undifferentiated carcinoma is a more challenging entity, both in terms of determining the primary site and in offering therapeutics options. Table 2 highlights the favourable and unfavourable subsets of CUP. In our opinion, it is the undifferentiated carcinoma which requires detailed molecular sequencing with larger panels, and indeed occasionally exomic and whole genome sequencing (see illustrative CUP case).

**Table 1:** Lineage-specific immunohistochemical markers (note: we highlight that as carcinomas become more undifferentiated, the differentiation markers (e.g. CDX2, Hep Par-1) become less reliable)

Origin	Recommended Immunostains	Comments
Colorectal	CK7-/CK20 + (66%), [4] CK7 + /CK20 + (15%) [4] or CK7 + /CK20- (2%), [4] CDX2 + (97%) [5]	CK7-/CK20 + in combination with CDX2 + is highly suggestive of colorectal origin CK7 + /CK20- and CDX2- profile are often associated with MMR deficient and or BRAF mutated colorectal carcinoma [6, 7]
Pancreaticobiliary and upper GI	Pancreas: CK7 + /CK20- (majority) [8] Biliary: CK7 + /CK20 + (majority) [9] Gastric: CK7 + /CK20- (majority) CDX2-/weak + , CK19 + , AE1/3 + , Hep Par-1-	CK7 + /CK20- in combination with CDX2- /weak + is suggestive of upper GI/pancreaticobiliary origin, however does not outrule primary lung origin Note: Gata3 + pancreatic adenocarcinoma (16%) [10]
Liver	Hep Par-1 + (95%), [11] CD34 + (sinusoidal capillarisation), glypican-3 + (90%), [12] glutamine synthetase + , HSP70 + , AE1/3-, CK19- (70%), CK7-/CK20- (90%)	Hep-par1 can be lost in poorly differentiated hepatocellular carcinoma [13] Note: CK7 + in fibrolamellar carcinoma [14]
Lung	Adenocarcinoma: TTF-1 (80%), Napsin A (80%), [11] CK7 + /CK20- Squamous cell carcinoma: p63 (87%) [15], p40 (77–100%) [16], CK5/6 (79%) [15], Gata3 + (12%) [17]	TTF-1 staining trumps all other stains and is highly suggestive of lung adenocarcinoma origin [18] Note: CK7 + /CK20 + lung mucinous adenocarcinoma (17%) [19]
Breast	Gata3 + (> 90%) [17], CK7 + /CK20- (> 90%), mammaglobin + (60%) [11], GCDFP + (60%) [11], ER/PR + (60%) [11]	Note: TTF-1 + breast carcinoma (2%) [20] and SOX10 + basal-like breast cancer (69%) [21] Note: mammaglobin + in mammary analogue secretory carcinoma of salivary gland
Prostate	PSA + (> 90%) [22], 50PSAP + (99%) [22], NKX3.1 + (80%) [22], Prostein + , CK7-, CK20- [23]	PSA can be lost in high grade adenocarcinoma [24] NKX3.1 has a high sensitivity and specificity but can be positive in male breast cancers [22]
Gynae tract	High grade serous: P53 -/ + (mutant pattern), p16 diffuse + , ER ± , Pax8 + Endometrioid: p53 wild type pattern, p16-, Pax8 + , ER + (> 90%) [11] Cervical adenocarcinoma: ER-, Pax8 + (70%) [25], HPV ish + , p16 diffuse +	CK7 + /CK20- in combination with Pax8 + is highly suggestive of gynae tract origin Note: CK7 + /CK20 + in primary mucinous tumours of ovary (74%) [26]
Urothelial	CK7 + /CK20 + (60%), [27] CK7 + /CK20- (30%) [28], p63 + , Gata3 + (> 90%) [17], uroplakin +	p63 is not specific for urothelium and is positive in squamous tumours from other sites
Neuroendocrine carcinomas (NEC)	Chromogranin A ± , CD56 + , synaptophysin + , AE1/3 + , SSTR2A ± Cytokeratin weak ±	While chromogranin A is most specific, poorly differentiated NECs are often negative [29] Synaptophysin is very sensitive but less specific [29] however is useful when Chromogranin A - While TTF-1 + in a well-differentiated NET favours primary site from lung/head and neck, less helpful in NECs as may express it regardless of primary site
Renal	RCC + , PAX8 + , 56CD10 + , CK7 ± , CK20-, AE1/3 +	CK7 + in papillary RCC type 1, clear cell papillary RCC and CK7- in clear cell RCC, papillary RCC type 2 [30]
Thyroid	TTF-1 + (> 90%) [31], thyroglobulin + , Pax8 + [32] CK7 + /CK20-, CK19 +	Medullary thyroid carcinoma shows a different profile: TTF-1 + , thyroglobulin-, Pax8 + , calcitonin + , chromogranin + , synaptophysin + [33]
Adrenal cortex	Melan-A + (94%) [34], inhibin + (92%) [35], calretinin + (96%) [35], CK7-/CK20-[23]	
Germ cell tumour	OCT3/4 + , SALL4 + , PLAP + , HCG + , AFP + , glypican3 + , CK7-/CK20- [23]	
Mesothelioma	Calretinin + (> 90%) [36], CK5/6 + (> 90%) [36], CK7 + , D2-40 + , WT-1 + (90%) [36], AE1/3 + , Gata3 + (58%) [17]

**Table 2:** Favourable and unfavourable subsets within carcinoma of unknown primary (CUP)

Favourable subsets	Unfavourable subsets
Adenocarcinoma with colonic profile (CK20 + , CK7-, CDX2 +)	Poorly differentiated carcinoma
Women with papillary adenocarcinoma of the peritoneal cavity	Adenocarcinoma metastatic to liver or other organs
Women with adenocarcinoma involving axillary lymph nodes	Non-papillary malignant ascites (adenocarcinoma)
Squamous cell carcinoma involving cervical lymph nodes	Multiple cerebral metastases (adenocarcinoma or squamous cell carcinoma)
Squamous cell carcinoma involving only inguinal lymph nodes	Multiple lung or pleural metastases (adenocarcinoma)
Poorly differentiated neuroendocrine carcinomas	Multiple metastatic bone disease (adenocarcinoma)
Men with blastic bone metastases and raised prostate specific antigen (PSA)	Squamous cell carcinoma of the abdominal cavity
Patients with a single small and potentially resectable tumour	

Adapted from [37]

Glandular morphology suggests a metastatic adenocarcinoma on H&E, with columnar cells with intracytoplasmic and/or extracellular mucin. Intestinal differentiation characterised by tall pseudostratified columnar epithelium with geographic necrosis suggests colorectal origin. In contrast, cuboidal to low columnar cells arranged in a single layer without nuclear pseudostratification or prominent necrosis may suggest pancreaticobiliary origin. The classic glandular pattern of an adenocarcinoma can also be seen in some solid organ tumours, germ cell tumours, and mesotheliomas; therefore, caution must be exercised. Features suggesting squamous differentiation include cohesive islands of cells with eosinophilic cytoplasm, intercellular bridges, and keratin pearls. Squamous carcinoma can then be further classified, using p16, as HPV or non-HPV related which can guide management. Squamoid morphology is not only seen in squamous carcinomas but can be seen in urothelial/transitional, basal, and some adenocarcinomas. Neuroendocrine tumours are grouped as well or poorly differentiated regardless of primary site, while grading varies according to tissue of origin (ToO). Well-differentiated neuroendocrine tumours show characteristic organoid growth with nests/trabeculae of centrally placed, uniform, round cells with coarse “salt and pepper” chromatin. Poorly differentiated neuroendocrine carcinomas which are more likely in CUP, either show small cell morphology with solid sheets of cells with hyperchromatic nuclei and scant cytoplasm or large cell morphology with vesicular nuclei and prominent nucleoli [3, 29].

A variety of other morphologic patterns can also be encountered such as epithelioid, small round blue, plasmacytoid, spindled, or undifferentiated morphology. Epithelioid morphology is characterised by polygonal cells with abundant cytoplasm, round to oval nuclei, and distinct nucleoli and is suggestive of a carcinoma; however, this morphology can also be seen in melanomas and sarcomas. In contrast, sarcomatoid morphology comprises fascicles of spindled cells with elongated nuclei. Metastatic sarcomatoid carcinoma or melanoma is more common in CUP than metastatic sarcoma. Sarcomatoid differentiation is particularly common in squamous tumours and carcinoma of the breast, kidney, bladder, and germ cell tumours. Of note, identification of intracytoplasmic lumina and red blood cell extravasation is helpful to suggest vascular origin (e.g. metastatic angiosarcoma) in tumours which may show predominant spindled or epithelioid morphology [3].

## Appropriate IHC and diagnostic pitfalls

Tumours can usually be broadly classified as a carcinoma rather than melanoma, lymphoma, or sarcoma on the first H&E. An initial broad panel can be performed if necessary and only if abundant tissue has been provided; however, this is not required in the majority of cases. In the era of precision oncology, preference however should be given to retaining tumour tissue for predictive testing, either IHC (e.g. MMR, PDL-1) or molecular (panel testing or WES). Our practice has evolved to obtaining a limited IHC panel with additional discussion advised at multidisciplinary meeting to retain sufficient tumour tissue for predictive testing. If classified as a carcinoma, a tumour can be further subtyped as squamous (p63, CK5/6), urothelial (Gata3, uroplakin), neuroendocrine (synaptophysin, chromogranin), solid organ (hepatocellular, HepPar-1, glypican 3; renal cell, RCC, Pax8, CD10; thyroid, TTF-1, thyroglobulin, Pax8), or adenocarcinoma. If it is an adenocarcinoma, likely site of origin can next be suggested using other markers in a lineage-specific panel (Table 1). The most recent NICE guidelines include CK7, CK20, TTF-1, ER/PR (female), or PSA (male) [38]. Antibodies should be examined in panels rather than in isolation, and the decision on which to choose will be made by the individual histopathologist, guided by clinical symptoms, serology, and radiological findings suggesting the most likely primary site. Consideration of age is vital as children develop very different tumours, and the pathologist will have alternative differential diagnoses in mind. Males and females develop tumours at varying primary sites also; therefore, immunopanels performed will also vary and have different clinical significance. WT-1 expression in a female patient may suggest an ovarian serous carcinoma for example, while mesothelioma would be favoured in a male. Final diagnosis should never depend on immunohistochemistry alone. It must be made in the correct clinicopathological context [3, 38].

Some diagnostic difficulties to remain conscious of in terms of IHC include that the more poorly differentiated a tumour is, the less likely it is to express tissue antigens. It is also important to recognise that tumours are heterogeneous and a single biopsy may not be representative of an entire tumour. There is no current international consensus on the optimal pathological approach; however, current European/US guidelines recommend a step-wise strategy [3, 39, 40]. In terms of the staining itself, problems can occur when no staining is seen or where prominent necrosis is present affecting interpretation [2].

**Fig. 3:**
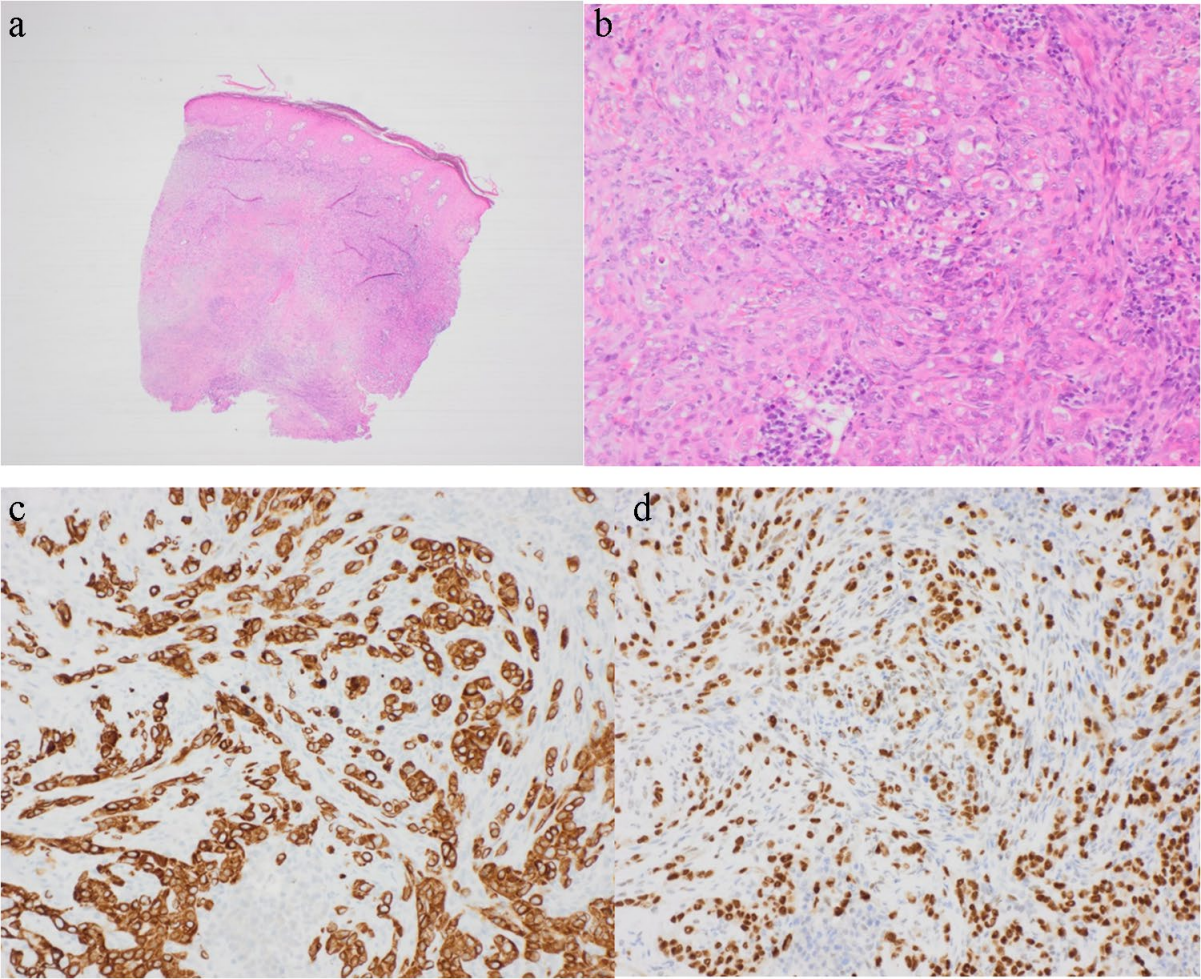
Metastatic epithelioid angiosarcoma. **a** Low power view and **b** high power view of skin H&E showing an epithelioid tumour with vasoformative foci. **c** Strong AE1/3 expression. **d** Strong nuclear ERG expression

Unusual staining patterns can also cause confusion, for example, a metastatic colorectal adenocarcinoma not staining with the expected CK20 + /CK7- profile, but which is consistent with metastases when staining and morphology is similar in the primary tumour. Expression of cytokeratin may be also be misleading. An example would be a tumour with epithelioid morphology and strong cytokeratin expression making metastatic carcinoma a strong consideration; however, recognition of vasoformative foci on H&E and addition of ERG immunohistochemistry will confirm metastatic epithelioid angiosarcoma, keratin positivity being a diagnostic pitfall if unaware of this particular staining profile (Fig. 3). Other mesenchymal tumours occasionally expressing cytokeratins include epithelioid leiomyosarcomas and epithelioid hemangioendotheliomas. Keratins may also be expressed in tumours with evidence of epithelial differentiation, such as synovial sarcomas, myoepithelial carcinomas, and desmoplastic small round cell tumours, among others. Aberrant expression of epithelial markers has similarly been reported in melanomas with epithelioid morphology, and conversely, loss of conventional melanocytic marker expression has been reported in metastatic lesions, evidently requiring careful exclusion of other tumour types.

Other immunostains to consider include markers of responsiveness to immunotherapy such as mismatch repair (MMR) and PD-L1 immunohistochemistry. In terms of MMR status, it should be noted that four separate sections of tissue are required for four different antibodies: MLH1, MSH2, MSH6, and PMS2. If loss of protein expression is seen, tissue is then sent for further BRAF or hypermethylation testing depending on the likely primary tumour type [41]. PD-L1 IHC is carried out on samples with at least 100 viable tumour cells present on H&E. This test requires two separate sections of tissue, one for the stain and one for the negative control. Unfortunately, as part of the clinical development of currently approved PD-L1 inhibitors, separate assays were developed in parallel for each as companion biomarkers. Therefore, there is considerable variability in testing, with each inhibitor having its own primary antibody clone, detection reagents, staining platform, and cut-offs for positivity. This is therefore a difficult marker for the pathologist to score and requires specific training and experience [42, 43].

IHC such as NUT, SMARCA4, and INI-1 antibodies act as specific surrogates for molecular mutations in certain neoplasms, often poorly differentiated and presenting as CUP. Diffuse expression with characteristic speckled pattern seen on NUT IHC confirms the diagnosis of NUT carcinoma [44]. The loss of expression of SMARCA4 IHC is 85–100% specific for ovarian small cell carcinoma, hypercalcaemic type (SCCOHT) a rare but aggressive ovarian neoplasm. [45] INI-1 deficient sinonasal carcinoma shows loss of INI-1 IHC expression in 100% of cases along with 100% of renal medullary carcinomas and 98% of malignant rhabdoid tumours of the kidney, soft tissue, and CNS so while not entirely specific can help guide diagnosis. [46]

## Precision oncology

The traditional cancer classification paradigm of tissue and organ type is changing as more cancers are now subclassified by their molecular characteristics with the aim of providing personalised treatment strategies. In cases of suspected CUP, it is good practice to conserve material for molecular analysis, and in cases with limited tumour tissue, this might be more appropriate than extensive diagnostic efforts in cases that remain morphologically unclear. A variety of sample types can be analysed including cell culture, body fluids, and fresh or fixed tissue, each having various advantages and disadvantages. Many different molecular techniques are available ranging from simple, fast assays that interrogate a single area to large, complex assays that can sequence the entire genome. Sequencing and genomic technologies can help to identify actionable mutations and clarify therapeutic options for patients with CUP. Further informative molecular features like methylation status and gene expression may also be relevant in the context of CUP. The aim is to not only improve survival rates when compared with current empiric treatment but also to reduce unnecessary side effects from ineffective drugs and provide a more complete diagnosis [47]. It is likely that molecular profiling will be of more benefit, in terms of diagnostic and predictive power, than their more differentiated CUP counterparts.

There are few commercial diagnostic tests based on genomic methods: The EPICUP CE-IVD marked assay is based on Illumina’s 450 k methylation array [48]. It is the only approved ToO test within the European Union. In a comprehensive validation, EPICUP is shown to have 99.7% specificity and 97.7% sensitivity in 7691 tumours. In 174/181 (96%) of patients diagnosed by pathological examination and EPICUP, the same ToO was found. CancerTYPE ID assay is a 92-gene RT-qPCR assay commercialised by BioTheranostics [49]. It is not approved by the FDA. Pathworks/cancer type uses microarray and has been FDA approved, but little information is available from current owners of the test Vyant Bio [37]. Another test, SuperDX, is not commercialised but is indicative of potential for Nanostring nCounter technology [50]. In terms of the current paradigm of actionable mutations, the CUPISCO phase 2 trial (NCT03498521; Roche) ongoing from 2018 uses the Foundation One panel on formalin-fixed material to determine suitability for a range of personalized treatments. This is then randomized against standard chemotherapy. Finally, there are many research studies of ToO methods employing RNA/DNA sequencing data (for a full review, refer to Table 1 from Rassy et al.) [51].

The theoretical framework of all methods rely on the same fundamental principle: chromatin accessibility broadly defines cell states [52]. Simply put, genomic regions are open or closed to transcription, and so data on gene expression may reveal ToO. The relationship of chromatin structure to DNA methylation in cancer is still being resolved, but generally methylation patterns from tissues appear similar, and EPICUP leverages this [53]. Chromatin accessibility may act as a record of cell type because it allows mutations to accrue in open regions of the genome, and this ‘topological distribution’ of mutations can then be used to determine primary cell type. An added benefit of this approach versus RNA and methylation is that somatic driver mutations can be used to guide therapy, and so there is potentially a dual utility of WGS in the context of CUP.

## Mutational signatures

The mutational profile of cancer reflects the aberrant functioning and processes of the neoplastic cells. These aberrant processes leave mutational signatures which reflect the presence and effect of previous mutagenic exposures, both exogenous and endogenous, during the evolution of a tumour. Some signatures are strongly associated with environmental exposures, for example, signature 4 with tobacco smoking, while signature 7 is strongly associated with UV radiation. The presence of these signatures can be used as useful indicators of the primary site of the tumour, for example, signature 4 associated with lung primary, while the presence of signature 7 is associated with cutaneous primary. These mutational signatures can be detected by whole exome sequencing (WES) or whole genome sequencing (WGS); however, in clinical practice, this is not practical as most clinical assays perform targeted sequencing. Formalin fixed tissue poses another challenge in the analysis and detection of mutational signatures due to artefacts that are present in addition to mutations from other mutagenic exposures. While this is not currently used in clinical practice, it is an interesting and emerging area. [54, 55]

## Clinical management

While cachexia and weight loss are the most common symptoms of CUP patients, the initial clinical presentation depends on the organs involved by metastatic disease. Paraneoplastic syndromes may also occur and can manifest before a definitive diagnosis. Their timely recognition may lead to an earlier cancer diagnosis which might substantially alter the prognosis of CUP patients [56].

While the primary site is unknown, CUP can be classified into two subgroups (Table 2) to aid in therapeutic decision making. Ten to 15% are designated as clinically favourable including patients with good performance status and normal lactate dehydrogenase (LDH) level. [57] These patients have a median life expectancy of one year. This group should be treated similarly to patients with equivalent known primary tumours with metastases as retrospective analysis has shown that this subset of patients has a similar outcome in terms of clinical behaviour, tumour biology, disease response, and outcome [39]. Thirty to 60% should achieve long-term disease control with median survival increasing to 15–20 months on chemotherapy [58]. The vast majority of patients with CUP (85–90%) fall within the unfavourable subgroup, however, and have a poor prognosis with median survival of 4–9 months. These figures have remained largely unchanged despite the recent development of new chemotherapeutic agents [58]. Realistic treatment aims include modest improvement in survival, palliation of symptoms, and preservation of quality of life with chemotherapy or supportive measures [39].

Empiric systemic chemotherapy is considered the standard treatment for metastatic CUP, although improvements in overall survival are modest and unpredictable, particularly in patients with unfavourable factors [59]. Treatment decisions must incorporate patient’s wishes, performance status, and overall prognosis whilst enrolment in clinical trials is strongly encouraged. Initiating psychosocial support and palliative care early in the disease course is essential given the uncertain natural history. The choice of chemotherapy regimen is primarily influenced by histological classification. Importantly, patients in whom primary anatomic site is strongly suggested by clinical/pathologic criteria should no longer be considered CUP, and treatment should proceed in accordance with established clinical guidelines for that tumour type.

Treatment guidelines for the most common subtype, adenocarcinoma, remain general in nature, extrapolated from other tumours including lung, ovarian, and gastric carcinoma. The most commonly employed regimen is a combination of carboplatin-paclitaxel, based largely on evidence from a handful of phase II/III trials [60, 61]. Alternative doublet regimens include cisplatin-gemcitabine and capecitabine-oxaliplatin which may be selected based on performance status, physician preference, and side-effect profile [62, 63]. Squamous carcinoma in the absence of an obvious primary represents a minority of CUP; however, level of evidence that exists to support systemic chemotherapy is very low. Historically, the most frequently used combination regimen is cisplatin-fluorouracil, based on longstanding use in anal, head and neck, and oesophageal cancers [64]. It has the advantage of compatibility with concurrent radiotherapy. As mentioned, NGS has the power to identify genomic aberrations within CUP tumours that may be targeted therapeutically, and studies have reported wide ranges of actionable mutations from 30 to 85% [65]. Similarly, immunotherapy is also emerging in several cancer types, with promising response rates seen in patients with metastatic disease and may be an option for a minority of patients with CUP. Pembrolizumab, an anti-PD1 monoclonal antibody, was the first tissue-/site-agnostic drug approved by the FDA in the second-line setting for metastatic tumours deficient in MMR proteins or high in microsatellite instability. Whilst there is no high level evidence, pembrolizumab should be strongly considered for these tumours, although they represent less than 2% of CUP [66].

## Illustrative CUP case

**Fig. 4:**
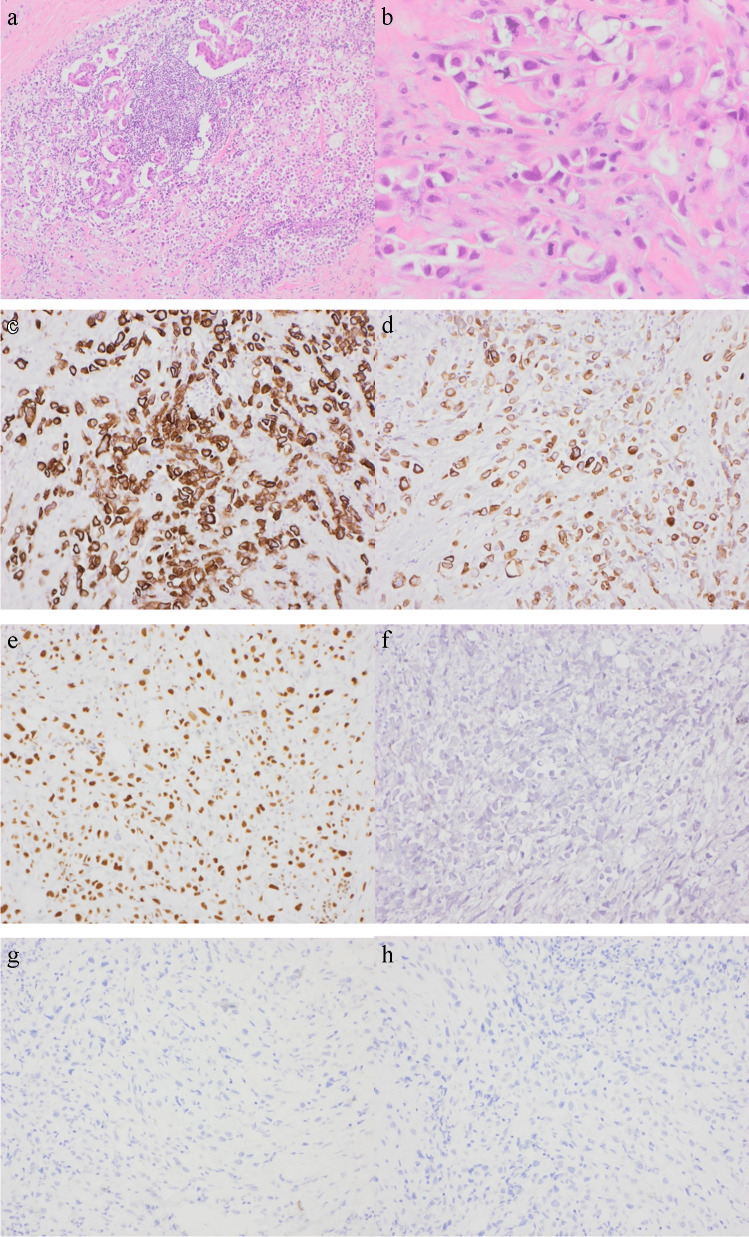
Illustrative case of metastatic CUP. **A** Low power glandular morphology. **B** High power poorly differentiated. **C** CK7 positive. **D** CK20 positive. **E** Gata 3 positive. **F** TTF1 negative. **G** CDX2 negative. **H** PSA negative

A middle age male presented with groin lymph node involvement by CUP. Extensive clinical, radiological, and IHC work-up revealed no definite primary site. Figure 4 shows H&E and relevant IHC panel results (CK7, 20, GATA3 positive with negative CDX2, TTF1/Napsin, and PSA. Whole genome sequencing revealed a high Tumour Mutation Burden of 12 SNV/MB. No 'driver' pathogenic mutations, a high level of focal copy number alterations, and structural variants affecting ROS1, & NTRK1. None of these changes were clinically actionable in this patient who despite providing consent for WGS in a research setting unfortunately passed away prior to full genomic analysis. This also highlights the need to provide an efficient molecular pathway with reasonable turnaround times given the poor outcome and short time to relapse of many CUP patients. Interestingly, while there was no obvious primary in this patient, we conclude that the anatomical site (often overlooked as a clue) and the GATA-3 positivity might well have pointed toward a bladder primary and in fact chemotherapy was directed toward this at one point in the patient’s clinical course.

## Conclusion

In summary, the diagnosis of CUP remains challenging and requires a multidisciplinary team effort to identify the primary site. The roles of the pathologist in the diagnostic pathway include the control of pre-analytical conditions, assessment of sample adequacy, diagnosis of cancer, and evaluation of prognostic and predictive markers. Sparing of tissue for molecular analysis is critical as targetable mutations or indication of site of origin may be identified in some cases. An integrated diagnostic report is ideal, with results discussed at a forum such as a molecular tumour board and matched with targeted treatment. This highly specialized evolving area ultimately leads to personalized medicine and improved outcomes for patients by giving drugs that will specifically target their tumour and save the patient unnecessary side effects of empiric non targeted treatment [67]. Research and improved understanding of genomic variations driving tumour development is critically important for the development of new drug therapies. This approach will hopefully lead to improved clinical outcomes for patients with CUP and may help to enrol more of our Irish patients in international cancer clinical trials.

## Data Availability

Not applicable.
